# Crystal structures of two new carbazole derivatives: 12-(4-nitro­phen­yl)-7-phenyl­sulfonyl-7*H*-benzofuro[2,3-*b*]carbazole and 2-methyl-4-(4-nitro­phen­yl)-9-phenyl­sulfonyl-9*H*-thieno[2,3-*b*]carbazole

**DOI:** 10.1107/S2056989016016996

**Published:** 2016-11-04

**Authors:** K. Swaminathan, P. Narayanan, K. Sethusankar, Velu Saravanan, Arasambattu K. Mohanakrishnan

**Affiliations:** a Department of Physics, RKM Vivekananda College (Autonomous), Chennai 600 004, India; b Department of Organic Chemistry, University of Madras, Guindy Campus, Chennai 600 025, India

**Keywords:** crystal structure, carbazole derivatives, phenyl­sulfon­yl, benzo­furan, thio­phene, hydrogen bonding, C—H⋯π inter­actions, offset π–π inter­actions

## Abstract

The title compounds, C_30_H_18_N_2_O_5_S, (I), and C_27_H_18_N_2_O_4_S_2_, (II), are carbazole derivatives with a phenyl­sulfonyl group and a nitro­phenyl group attached to the carbazole moiety in identical positions in both mol­ecules. A benzo­furan ring system in (I) and a methyl­thio­phene ring in (II) are fused with the respective carbazole moieties on the same sides. The mean plane of the carbazole ring system makes a dihedral angle of 3.17 (7)° with the benzo­furan ring system in (I) and a dihedral angle of 3.39 (11)° with the methyl­thio­phene ring in (II), implying that both fused units are essentially planar. The mean planes of the carbazole ring systems in both the compounds are almost orthogonal to the respective nitro-substituted phenyl rings, making dihedral angles of 75.64 (10) and 77.63 (12)° in compounds (I) and (II), respectively. In (I), the phenyl­sulfonyl ring system is positionally disordered with a refined occupancy ratio of 0.63 (2):0.37 (2). In both compounds, the mol­ecular structures are stabilized by intra­molecular C—H⋯O hydrogen bonds, generating *S*(6) ring motifs with the sulfone group O atoms. In the crystal of compound (I), mol­ecules are linked by pairs of C—H⋯O hydrogen bonds, which generate *R*_2_^2^(18) inversion dimers, and inter­connected by *C*(14) chains running along the *c*-axis direction, whereas in compound (II), the C—H⋯O hydrogen bonds generate *R*_4_^3^(37) ring motifs. In the crystals of both compounds, C—H⋯O hydrogen-bonded sheets are formed lying parallel to (10-1). In addition, C—H⋯π and offset π–π inter­actions [inter­centroid distance = 3.7158 (14) Å in (I) and 3.9040 (15) Å in (II)] are also present in the crystals of both compounds.

## Abstract 2

The title compounds are carbazole derivatives, with a benzo­furan ring system in (I) and a methyl­thio­phene ring in (II) fused with the respective carbazole moiety. In the crystals of both compounds, mol­ecules are linked *via* C—H⋯O hydrogen bonds, forming sheets lying parallel to (10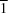).

## Chemical context   

Carbazole and its derivatives are inter­esting compounds owing to their applications in pharmacy and mol­ecular electronics. Carbazole derivatives exhibit various biological activities such as anti­tumor (Itoigawa *et al.*, 2000 ▸), anti-oxidative (Tachibana *et al.*, 2001 ▸), anti-inflammatory and anti­mutagenic (Ramsewak *et al.*, 1999 ▸). They also exhibit electroactivity and luminescence and are considered to be potential candidates for electronic applications, such as colour displays, organic semiconductors, laser and solar cells (Friend, *et al.* 1999 ▸). Tetra­hydro­carbazole systems are present in the framework of a number of indole-type alkaloids of biological inter­est (Saxton, 1983 ▸). Carbazole-based heterocyclic polymer systems can be chemically or electrochemically polymerized to give products with a number of applications, such as rechargeable batteries (Sacak, 1999 ▸) and electrochromic displays (Santhanam & Sundaresan, 1986 ▸). This enables their use as suitable building blocks for the design and synthesis of mol­ecular glasses, which are widely studied as components of electroactive and photoactive materials (Zhang *et al.*, 2004 ▸). Against this background, the X-ray structure determination of the title compounds, (I) and (II), has been carried out to study their structural aspects and the results are presented here.
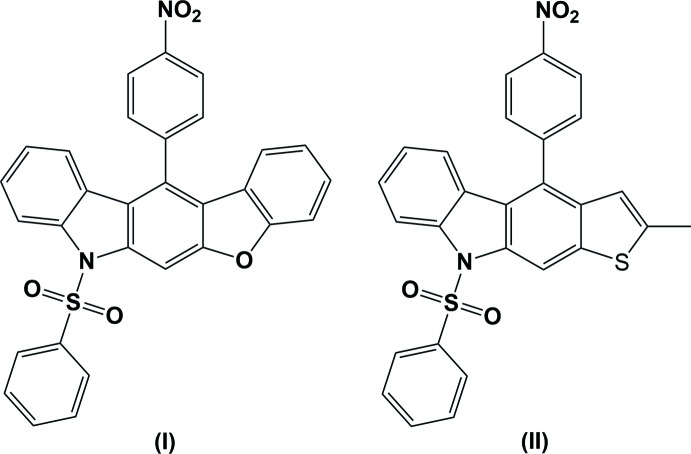


## Structural commentary   

**Figure 1:**
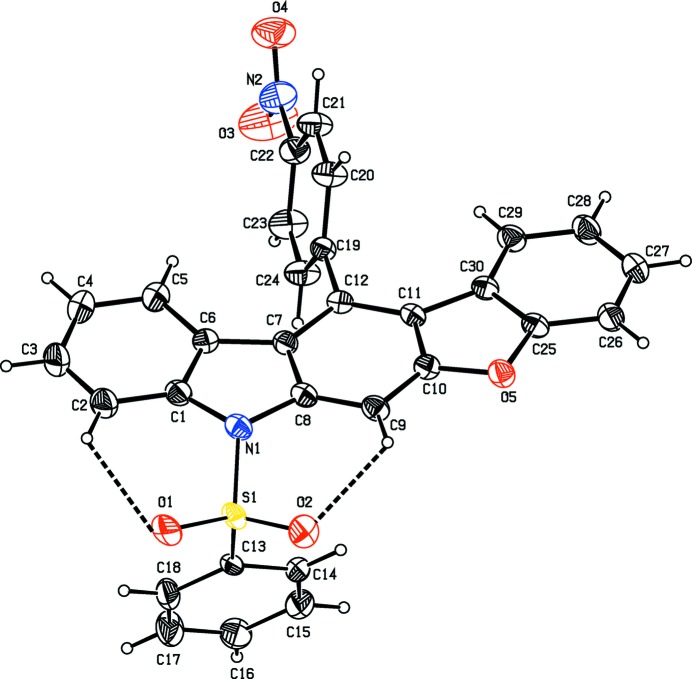
The mol­ecular structure of compound (I), showing the atom labelling. Displacement ellipsoids are drawn at the 30% probability level. Intra­molecular C2—H2⋯O1 and C9—H9⋯O2 hydrogen bonds, which generate two *S*(6) ring motifs, are shown as dashed lines (see Table 1 ▸). For the sake of clarity, the minor component of the disordered phenyl­sulfonyl ring has been omitted.

The mol­ecular structures of the title compounds, (I) and (II), are illustrated in Figs. 1 ▸ and 2 ▸, respectively. In both compounds, the carbazole ring systems (N1/C1–C12) are essentially planar with maximum deviations of 0.089 (3) and 0.089 (3) Å for atom C10 in compounds (I) and (II), respectively. In compound (I), the benzo­furan moiety (O5/C10/C11/CC25–C30) is essentially planar with a maximum deviation of 0.021 (3) Å for atom C10 while the phenyl­sulfonyl ring system is positionally disordered with a refined occupancy factor of 0.63 (2): 0.37 (2).

The mean planes of the carbazole ring systems make dihedral angles of 3.17 (7) and 3.39 (11)°, respectively, with the benzo­furan ring in (I) and the methyl­thio­phene ring in (II), indicating that the ring systems they are essentially coplanar. The nitro­phenyl rings in compounds (I) and (II) are inclined to the carbazole ring system by 75.64 (10) and 77.63 (12)°, respectively. The NO_2_ groups are inclined to the benzene ring (C19–C24) to which they are attached by 9.8 (4)° in (I) and 9.3 (3)° in (II). The phenyl­sulfonyl ring (C13–C18) is almost normal to the nitro-substituted phenyl ring (C19–C24) with a dihedral angle of 84.7 (2)° in (I) and 83.98 (17)° in (II).

In both compounds, as a result of the electron-withdrawing character of the phenyl­sulfonyl group, the N—C*sp* ^2^ bond lengths are longer than the mean value of 1.355 (14) Å for N—C bond lengths (CSD; Groom *et al.*, 2016 ▸). Atom S1 has a distorted tetra­hedral geometry. The widening of the O1=S1=O2 angle and narrowing of the N1—S1—C13 angle from the ideal tetra­hedral values are attributed to the Thorpe–Ingold effect (Bassindale, 1984 ▸). The widening of the angles may be due to the repulsive inter­action between the two short S=O bonds.

The sums of the bond angles around atom N1 are 349.58° in (I) and 351.18° in (II), intermediate between *sp* ^2^ and *sp* ^3^ hybridization. In both compounds, the mol­ecular structure is stabilized by intra­molecular C—H⋯O hydrogen bonds, which generate *S*(6) ring motifs with the sulfone oxygen atoms (Tables 1 ▸ and 2 ▸).

## Supra­molecular features   

**Figure 3:**
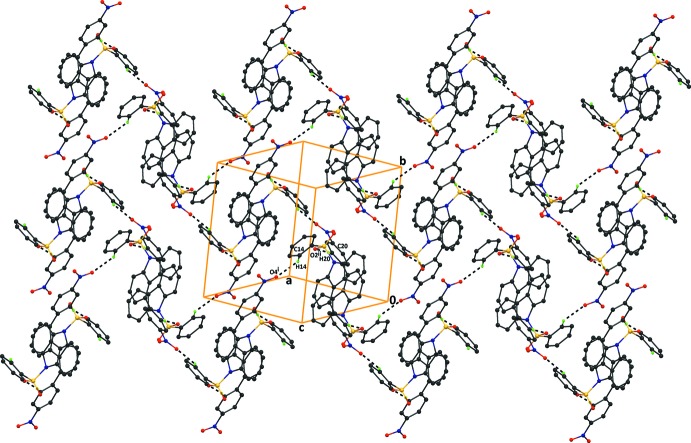
The crystal packing of compound (I), viewed normal to the (10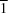) plane, showing C—H⋯O hydrogen bonds that generate 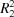
(18) inversion dimers and the C—H⋯O hydrogen bonds that generate *C*(14) chains running along the *c*-axis direction (see Table 1 ▸ for details). H atoms not involved in the hydrogen bonding and the benzo­furan ring have been excluded for clarity.

In the crystal of compound (I), mol­ecules are linked *via* pairs of C20—H20⋯O2^ii^ hydrogen bonds (Table 1 ▸), forming 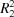
(18) inversion dimers. The mol­ecules are also inter­connected by C14—H14⋯O4^i^ hydrogen bonds, which generate *C*(14) chains. These inter­actions result in the formation of sheets parallel to (10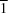). The crystal packing also features C16—H16⋯*Cg*1^iii^ and C23—H23⋯*Cg*3^iv^ inter­actions (Table 1 ▸ and Fig. 3 ▸; *Cg*1 and *Cg*3 are the centroids of the rings C10/C11/C25/30/O5 and C1–C6, respectively. There are also offset π–π inter­actions present [*Cg*4⋯*Cg*4(−*x* + 1, −*y* + 2, −*z*) = 3.7158 (14) Å, inter­planar distance = 3.472 (1) Å, slippage = 1.324 (11) Å; *Cg*4 is the centroid of the C7–C12 ring]; see Table 1 ▸ and Fig. 3 ▸.

**Figure 4:**
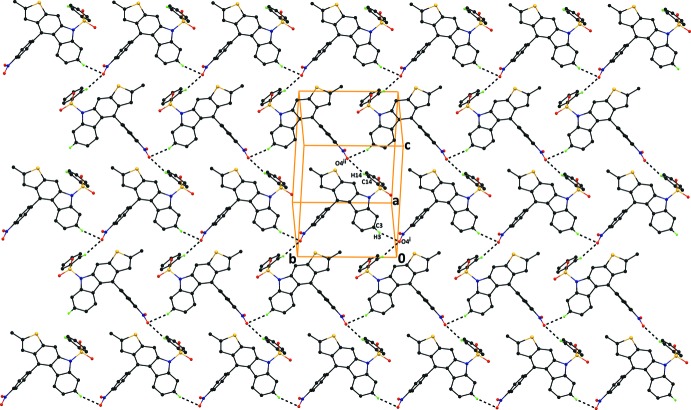
The crystal packing of compound (II), viewed along the *ac* diagonal, showing the inter­molecular C—H⋯O hydrogen bonds (see Table 2 ▸), which generate 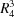
(37) ring motifs and form sheets lying parallel to the (10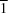) plane. H atoms not involved in hydrogen bonding have been excluded for clarity.

**Figure 5:**
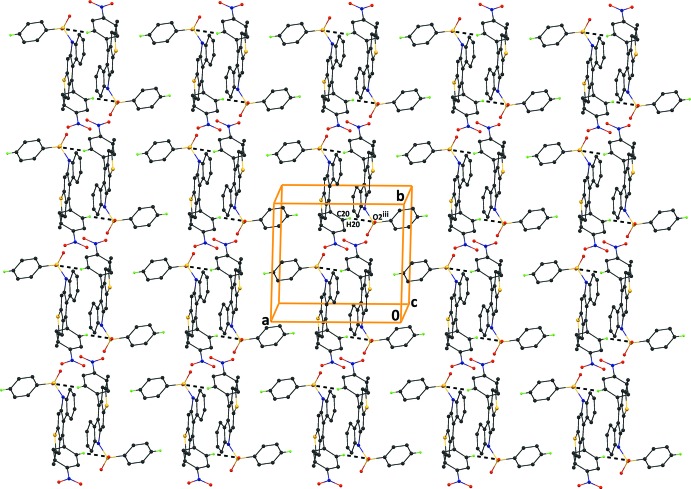
The crystal packing of compound (II), viewed along the *c* axis, showing the C—H⋯O inter­molecular hydrogen bonds which generate 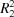
(18) inversion dimers (see Table 2 ▸). H atoms not involved in hydrogen bonding have been excluded for clarity.

In the crystal of compound (II), mol­ecules are linked by C3—H3⋯O4^i^ and C14—H14⋯O^ii^4 hydrogen bonds (Table 2 ▸), which result in the formation of 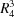
(37) ring motifs (Fig. 4 ▸). The crystal packing also features pairs of C20—H20⋯O2^iii^ hydrogen bonds, which generate 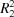
(18) inversion dimers (Fig. 5 ▸), which are inter­connected by C16—H16⋯*Cg*1^iv^ and C23—H23⋯*Cg*3^v^ inter­actions [*Cg*1 and *Cg*3 are the centroids of rings C10/C11/C25/C26/S2 and C1–C6, respectively]. These inter­actions result in the formation of sheets parallel to (10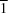). There are also offset π–π inter­actions present [*Cg*4⋯*Cg*4(−*x* + 1, −*y*, −*z* + 2) = 3.9040 (15) Å, inter­planar distance = 3.791 (1) Å, slippage 0.932 Å; *Cg*4 is the centroid of the C7–C12, ring]; see Table 2 ▸ and Figs. 4 ▸ and 5 ▸.

## Database survey   

A search of Cambridge Structural Database (CSD version 5.37; last update May 2016; Groom *et al.*, 2016 ▸) yielded four hits for 7*H*-[1] benzo­furan­[2,3-*b*]carbazole and 47 hits for 9-(phenyl­sulfon­yl)-9*H*-carbazole. However, the compound 7-phenyl­sulfonyl-7*H*-benzo­furan­[2,3-*b*]carbazole (EYOFEE01; Panchatcharam *et al.*, 2011 ▸), which crystallizes in *P*2_1_/*c* is the closest analogue of compound (I). The compound 2-methyl-9- (phenyl­sulfon­yl)-9*H*-thieno[2,3-*b*]carbazole (IQOBIA; Sureshbabu *et al.*, 2011 ▸), which crystallizes in space group *P*2_1_/*c*, is the closest analogue of compound (II). The crystal packing of the title compounds is stabilized by C—H⋯O, C—H⋯π and π–π inter­actions but the related structures (EYOFEE01; Panchatcharam *et al.*, 2011 ▸) exhibit C—H⋯π and π–π inter­actions only.

## Synthesis and crystallization   

**Compound (I)**: A solution of [3-(4-nitro­benzo­yl)-1-(phenyl­sulfon­yl)-1*H*-indol-2-yl]methyl pivalate (0.1 g, 1.92 mmol), anhydrous SnCl_4_ (0.06 g, 2.30 mmol) and benzo­furan (0.027 g, 2.30 mmol) in dry DCE (10 ml) was stirred at room temperature under nitro­gen for 3 h. After completion of the reaction (monitored by TLC), it was poured into ice–water (100 ml). The organic layer was separated and the aqueous layer was extracted with DCM (2 × 20 ml). The combined extract was washed with water (3 × 50ml) and dried (Na_2_SO_4_). Removal of the solvent by column chromatographic purification (silica gel; hexa­ne–ethyl acetate, 8:2) yielded compound (I) as a colourless solid (0.073 g, 74%). Colourless block-like crystals were obtained by slow evaporation of a solution of (I) in ethyl acetate at room temperature (m.p. 589–591 K).

**Compound (II)**: A solution of [3-(4-nitro­benzo­yl)-1-(phenyl­sulfon­yl)-*H*-indol-2-yl]methyl pivalate (0.1 g, 1.92 mmol), anhydrous SnCl_4_ (0.06 g, 2.30 mmol) and 2-methyl­thio­phene (0.024 g, 2.30 mmol) in dry DCE (10 ml) was stirred at room temperature under nitro­gen atmosphere for 3 h. After the completion of the reaction (monitored by TLC), it was poured into ice–water (100 ml), the organic layer was separated and the aqueous layer was extracted with DCM (2 × 20ml). The combined extract was washed with water (3 × 50 ml) and dried (Na_2_SO_4_). Removal of the solvent by column chromatographic purification (silica gel; hexa­ne–ethyl acetate, 8:2) yielded compound (II) as a colourless solid (0.067 g, 72%). Colourless block-like crystals were obtained by slow evaporation of a solution of (II) in ethyl acetate at room temperature (m.p. 531–533 K).

## Refinement   

Crystal data, data collection and structure refinement details for compounds (I) and (II) are summarized in Table 3 ▸. The positions of the hydrogen atoms were localized from the difference electron-density maps. The C-bound H atoms were treated as riding atoms: C—H = 0.93-0.96 Å with *U*_iso_(H)= 1.5*U*_eq_(C-meth­yl) and 1.2*U*_eq_(C) for other H atoms. In compound (I), the phenyl­sulfonyl ring (C13–C18) is positionally disordered with a refined occupancy ratio of 0.63 (2): 0.37 (2). The bond distances of the disordered components were restrained using standard similarity restraint SADI [*SHELXL97*; Sheldrick, 2008 ▸] with s.u. of 0.01Å. Ellipsoid displacement (SIMU and DELU) restraints were also applied to the disordered ring. The methyl groups were allowed to rotate, but not to tip, to best fit the electron density.

## Supplementary Material

Crystal structure: contains datablock(s) I, II, global. DOI: 10.1107/S2056989016016996/su5333sup1.cif

Structure factors: contains datablock(s) I. DOI: 10.1107/S2056989016016996/su5333Isup2.hkl

Structure factors: contains datablock(s) II. DOI: 10.1107/S2056989016016996/su5333IIsup3.hkl

Supporting information file. DOI: 10.1107/S2056989016016996/su5333Isup4.cml

Supporting information file. DOI: 10.1107/S2056989016016996/su5333IIsup5.cml

CCDC reference: 1473995

Additional supporting information:  crystallographic information; 3D view; checkCIF report

## Figures and Tables

**Figure 2:**
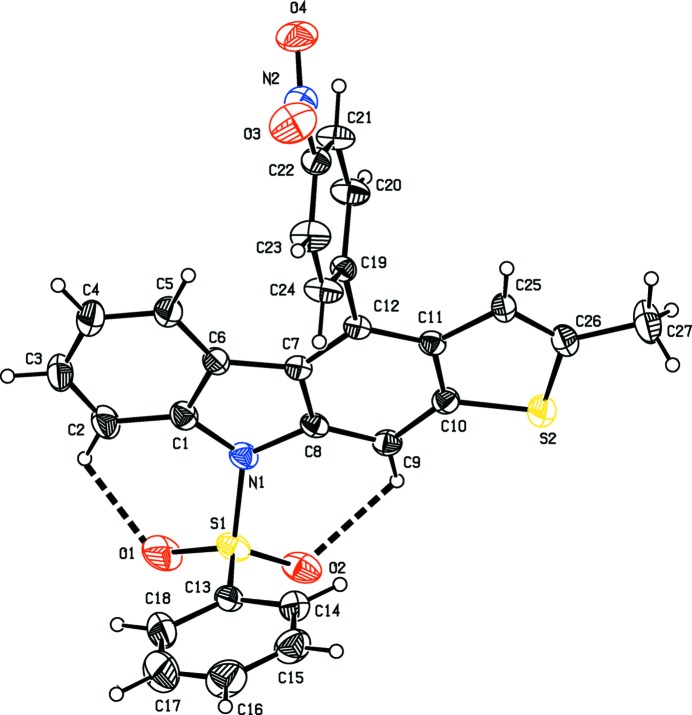
The mol­ecular structure of compound (II), showing the atom labelling. Displacement ellipsoids are drawn at the 30% probability level. Intra­molecular C2—H2⋯O1 and C9—H9⋯O2 hydrogen bonds, which generate two *S*(6) ring motifs, are shown as dashed lines (see Table 2 ▸).

**Table 1:** Hydrogen-bond geometry (Å, °) for (I) *Cg*1 is the centroid of the furan ring O5/C10/C11/C25/C30 and *Cg*3 is the centroid of the benzene ring C1–C6.

*D*—H⋯*A*	*D*—H	H⋯*A*	*D*⋯*A*	*D*—H⋯*A*
C2—H2⋯O1	0.93	2.41	3.015 (9)	122
C9—H9⋯O2	0.93	2.28	2.849 (9)	119
C14—H14⋯O4^i^	0.93	2.55	3.296 (7)	138
C20—H20⋯O2^ii^	0.93	2.53	3.454 (11)	172
C16—H16⋯*Cg*1^iii^	0.93	2.76	3.676 (6)	169
C23—H23⋯*Cg*3^iv^	0.93	2.99	3.908 (4)	169

Symmetry codes: (i) 
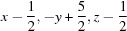
; (ii) 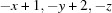; (iii) 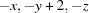; (iv) 
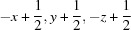
.

**Table 2:** Hydrogen-bond geometry (Å, °) for (II) *Cg*1 is the centroid of the thio­phene ring S2/C10/C11/C25/C26 and *Cg*3 is centroid of the benzene ring C1–C6.

*D*—H⋯*A*	*D*—H	H⋯*A*	*D*⋯*A*	*D*—H⋯*A*
C2—H2⋯O1	0.93	2.38	2.970 (4)	121
C9—H9⋯O2	0.93	2.33	2.907 (3)	120
C3—H3⋯O4^i^	0.93	2.48	3.356 (4)	157
C14—H14⋯O4^ii^	0.93	2.58	3.335 (4)	139
C20—H20⋯O2^iii^	0.93	2.53	3.368 (5)	150
C16—H16⋯*Cg*1^iv^	0.93	2.89	3.812 (4)	172
C23—H23⋯*Cg*3^v^	0.93	2.75	3.646 (4)	163

Symmetry codes: (i) 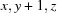; (ii) 
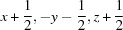
; (iii) 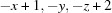; (iv) 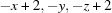; (v) 
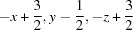
.

**Table 3:** Experimental details

	(I)	(II)
Crystal data		
Chemical formula	C_30_H_18_N_2_O_5_S	C_27_H_18_N_2_O_4_S_2_
*M*_r_	518.52	498.55
Crystal system, space group	Monoclinic, *P*2_1_/*n*	Monoclinic, *P*2_1_/*n*
Temperature (K)	296	296
*a*, *b*, *c* (Å)	12.1347 (5), 12.0708 (5), 17.6391 (7)	12.5052 (8), 11.2594 (6), 17.0731 (9)
β (°)	108.617 (2)	102.914 (2)
*V* (Å^3^)	2448.50 (17)	2343.1 (2)
*Z*	4	4
Radiation type	Mo *K*α	Mo *K*α
μ (mm^−1^)	0.18	0.27
Crystal size (mm)	0.25 × 0.20 × 0.10	0.35 × 0.30 × 0.25

Data collection		
Diffractometer	Bruker Kappa APEXII CCD	Bruker Kappa APEXII CCD
Absorption correction	Multi-scan (*SADABS*; Bruker, 2008 ▸)	Multi-scan (*SADABS*; Bruker, 2008 ▸)
*T*_min_, *T*_max_	0.958, 0.982	0.911, 0.936
No. of measured, independent and observed [*I* > 2σ(*I*)] reflections	32749, 4318, 2814	44472, 5100, 3714
*R*_int_	0.040	0.034
(sin θ/λ)_max_ (Å^−1^)	0.595	0.639

Refinement		
*R*[*F* ^2^ > 2σ(*F* ^2^)], *wR*(*F* ^2^), *S*	0.043, 0.131, 1.05	0.055, 0.163, 1.08
No. of reflections	4318	5100
No. of parameters	401	317
No. of restraints	140	0
H-atom treatment	H-atom parameters constrained	H-atom parameters constrained
Δρ_max_, Δρ_min_ (e Å^−3^)	0.28, −0.21	0.44, −0.36

Computer programs: *APEX2* and *SAINT* (Bruker, 2008 ▸), *SHELXS97* and *SHELXL97* (Sheldrick, 2008 ▸), *ORTEP-3 for Windows* (Farrugia, 2012 ▸), *Mercury* (Macrae *et al.*, 2008 ▸) and *PLATON* (Spek, 2009 ▸).

## supplementary crystallographic information

### (I) 12-(4-Nitrophenyl)-7-phenylsulfonyl-7H-benzofuro[2,3-b]carbazole Crystal data

**Table:**

C_30_H_18_N_2_O_5_S	*F*(000) = 1072
*M**_r_* = 518.52	*D*_x_ = 1.407 Mg m^−^^3^
Monoclinic, *P*2_1_/*n*	Mo *K*α radiation, λ = 0.71073 Å
Hall symbol: -P 2yn	Cell parameters from 4318 reflections
*a* = 12.1347 (5) Å	θ = 2.4–25.0°
*b* = 12.0708 (5) Å	µ = 0.18 mm^−^^1^
*c* = 17.6391 (7) Å	*T* = 296 K
β = 108.617 (2)°	Block, colourless
*V* = 2448.50 (17) Å^3^	0.25 × 0.20 × 0.10 mm
*Z* = 4	

### (I) 12-(4-Nitrophenyl)-7-phenylsulfonyl-7H-benzofuro[2,3-b]carbazole Data collection

**Table:**

Bruker Kappa APEXII CCD diffractometer	4318 independent reflections
Radiation source: fine-focus sealed tube	2814 reflections with *I* > 2σ(*I*)
Graphite monochromator	*R*_int_ = 0.040
ω & φ scans	θ_max_ = 25.0°, θ_min_ = 2.4°
Absorption correction: multi-scan (SADABS; Bruker, 2008)	*h* = −14→11
*T*_min_ = 0.958, *T*_max_ = 0.982	*k* = −14→14
32749 measured reflections	*l* = −18→20

### (I) 12-(4-Nitrophenyl)-7-phenylsulfonyl-7H-benzofuro[2,3-b]carbazole Refinement

**Table:**

Refinement on *F*^2^	Primary atom site location: structure-invariant direct methods
Least-squares matrix: full	Secondary atom site location: difference Fourier map
*R*[*F*^2^ > 2σ(*F*^2^)] = 0.043	Hydrogen site location: inferred from neighbouring sites
*wR*(*F*^2^) = 0.131	H-atom parameters constrained
*S* = 1.05	*w* = 1/[σ^2^(*F*_o_^2^) + (0.0594*P*)^2^ + 0.7253*P*] where *P* = (*F*_o_^2^ + 2*F*_c_^2^)/3
4318 reflections	(Δ/σ)_max_ = 0.002
401 parameters	Δρ_max_ = 0.28 e Å^−^^3^
140 restraints	Δρ_min_ = −0.21 e Å^−^^3^

### (I) 12-(4-Nitrophenyl)-7-phenylsulfonyl-7H-benzofuro[2,3-b]carbazole Special details

**Table:**

Geometry. All esds (except the esd in the dihedral angle between two l.s. planes) are estimated using the full covariance matrix. The cell esds are taken into account individually in the estimation of esds in distances, angles and torsion angles; correlations between esds in cell parameters are only used when they are defined by crystal symmetry. An approximate (isotropic) treatment of cell esds is used for estimating esds involving l.s. planes.
Refinement. Refinement of F^2^ against ALL reflections. The weighted R-factor wR and goodness of fit S are based on F^2^, conventional R-factors R are based on F, with F set to zero for negative F^2^. The threshold expression of F^2^ > 2sigma(F^2^) is used only for calculating R-factors(gt) etc. and is not relevant to the choice of reflections for refinement. R-factors based on F^2^ are statistically about twice as large as those based on F, and R- factors based on ALL data will be even larger.

### (I) 12-(4-Nitrophenyl)-7-phenylsulfonyl-7H-benzofuro[2,3-b]carbazole Fractional atomic coordinates and isotropic or equivalent isotropic displacement parameters (Å^2^)

**Table:**

	*x*	*y*	*z*	*U*_iso_*/*U*_eq_	Occ. (<1)
C8	0.36384 (19)	0.94124 (18)	0.02090 (14)	0.0501 (6)	
C9	0.3369 (2)	0.96522 (19)	−0.05953 (15)	0.0552 (6)	
H9	0.3249	0.9108	−0.0987	0.066*	
C10	0.3295 (2)	1.07565 (19)	−0.07650 (14)	0.0512 (6)	
C25	0.2981 (2)	1.22953 (19)	−0.14680 (14)	0.0540 (6)	
C26	0.2698 (2)	1.3006 (2)	−0.21190 (16)	0.0669 (7)	
H26	0.2531	1.2750	−0.2641	0.080*	
C27	0.2680 (2)	1.4115 (2)	−0.19423 (18)	0.0729 (8)	
H27	0.2483	1.4631	−0.2356	0.088*	
C28	0.2947 (2)	1.4475 (2)	−0.11625 (17)	0.0721 (8)	
H28	0.2932	1.5231	−0.1065	0.087*	
C29	0.3236 (2)	1.3757 (2)	−0.05248 (16)	0.0621 (7)	
H29	0.3418	1.4018	−0.0003	0.075*	
C30	0.32498 (19)	1.26319 (18)	−0.06814 (14)	0.0497 (6)	
C11	0.34632 (18)	1.16109 (17)	−0.02110 (13)	0.0465 (5)	
C12	0.37395 (18)	1.13576 (18)	0.06066 (13)	0.0480 (5)	
C7	0.38385 (18)	1.02299 (18)	0.08091 (13)	0.0488 (6)	
C6	0.4188 (2)	0.9654 (2)	0.15713 (15)	0.0558 (6)	
C5	0.4579 (3)	1.0012 (3)	0.23615 (17)	0.0798 (8)	
H5	0.4621	1.0765	0.2480	0.096*	
C4	0.4905 (3)	0.9234 (3)	0.29710 (19)	0.0967 (10)	
H4	0.5165	0.9465	0.3502	0.116*	
C3	0.4845 (3)	0.8114 (3)	0.2793 (2)	0.0917 (10)	
H3	0.5059	0.7604	0.3210	0.110*	
C2	0.4480 (2)	0.7735 (2)	0.20217 (19)	0.0751 (8)	
H2	0.4449	0.6981	0.1908	0.090*	
C1	0.4157 (2)	0.8517 (2)	0.14180 (15)	0.0575 (6)	
C19	0.38864 (19)	1.22457 (18)	0.12135 (13)	0.0501 (6)	
C20	0.4853 (2)	1.2920 (2)	0.14172 (16)	0.0698 (8)	
H20	0.5419	1.2810	0.1173	0.084*	
C21	0.5000 (2)	1.3751 (2)	0.19725 (17)	0.0749 (8)	
H21	0.5664	1.4190	0.2114	0.090*	
C22	0.4151 (2)	1.3916 (2)	0.23098 (15)	0.0656 (7)	
C23	0.3172 (3)	1.3273 (3)	0.21208 (18)	0.0837 (9)	
H23	0.2601	1.3400	0.2358	0.100*	
C24	0.3051 (2)	1.2436 (2)	0.15732 (17)	0.0726 (8)	
H24	0.2393	1.1989	0.1443	0.087*	
N1	0.38211 (16)	0.83465 (15)	0.05764 (12)	0.0563 (5)	
N2	0.4307 (3)	1.4799 (2)	0.29103 (16)	0.0916 (8)	
O5	0.30118 (15)	1.11572 (13)	−0.15385 (9)	0.0620 (5)	
O3	0.3507 (3)	1.5024 (3)	0.3147 (2)	0.1593 (14)	
O4	0.5242 (3)	1.5253 (2)	0.31592 (15)	0.1177 (9)	
S1	0.3009 (3)	0.72479 (18)	0.01555 (15)	0.0506 (10)	0.63 (2)
O1	0.3496 (10)	0.6265 (6)	0.0582 (5)	0.0755 (19)	0.63 (2)
O2	0.2837 (9)	0.7347 (7)	−0.0686 (2)	0.072 (2)	0.63 (2)
C13	0.1670 (5)	0.7463 (4)	0.0295 (4)	0.0473 (17)	0.63 (2)
C14	0.0952 (6)	0.8257 (4)	−0.0189 (4)	0.0530 (15)	0.63 (2)
H14	0.1180	0.8613	−0.0583	0.064*	0.63 (2)
C15	−0.0106 (5)	0.8520 (5)	−0.0086 (4)	0.0750 (19)	0.63 (2)
H15	−0.0586	0.9052	−0.0410	0.090*	0.63 (2)
C16	−0.0447 (4)	0.7989 (7)	0.0502 (4)	0.086 (2)	0.63 (2)
H16	−0.1154	0.8164	0.0572	0.103*	0.63 (2)
C17	0.0271 (5)	0.7194 (8)	0.0987 (4)	0.090 (3)	0.63 (2)
H17	0.0043	0.6839	0.1380	0.108*	0.63 (2)
C18	0.1329 (5)	0.6931 (6)	0.0883 (4)	0.075 (2)	0.63 (2)
H18	0.1809	0.6400	0.1207	0.090*	0.63 (2)
S1'	0.3118 (8)	0.7228 (6)	0.0149 (6)	0.102 (3)	0.37 (2)
O1'	0.379 (2)	0.6375 (16)	0.0646 (13)	0.133 (7)	0.37 (2)
O2'	0.2987 (18)	0.7264 (11)	−0.0681 (7)	0.095 (4)	0.37 (2)
C13'	0.1784 (11)	0.7417 (13)	0.0298 (10)	0.094 (5)	0.37 (2)
C14'	0.1010 (14)	0.8274 (11)	−0.0020 (12)	0.094 (4)	0.37 (2)
H14'	0.1214	0.8835	−0.0311	0.112*	0.37 (2)
C15'	−0.0070 (11)	0.8293 (15)	0.0097 (12)	0.115 (5)	0.37 (2)
H15'	−0.0588	0.8867	−0.0116	0.138*	0.37 (2)
C16'	−0.0375 (10)	0.746 (2)	0.0533 (10)	0.145 (6)	0.37 (2)
H16'	−0.1098	0.7468	0.0611	0.174*	0.37 (2)
C17'	0.0399 (14)	0.660 (2)	0.0851 (10)	0.151 (6)	0.37 (2)
H17'	0.0195	0.6037	0.1142	0.181*	0.37 (2)
C18'	0.1478 (12)	0.6579 (17)	0.0734 (11)	0.139 (5)	0.37 (2)
H18'	0.1996	0.6005	0.0947	0.167*	0.37 (2)

### (I) 12-(4-Nitrophenyl)-7-phenylsulfonyl-7H-benzofuro[2,3-b]carbazole Atomic displacement parameters (Å^2^)

**Table:**

	*U*^11^	*U*^22^	*U*^33^	*U*^12^	*U*^13^	*U*^23^
C8	0.0494 (13)	0.0414 (12)	0.0632 (16)	−0.0007 (10)	0.0232 (11)	0.0000 (11)
C9	0.0664 (16)	0.0426 (13)	0.0613 (16)	−0.0044 (11)	0.0270 (12)	−0.0069 (11)
C10	0.0543 (14)	0.0498 (14)	0.0523 (15)	−0.0023 (11)	0.0211 (11)	−0.0048 (11)
C25	0.0598 (15)	0.0401 (13)	0.0647 (16)	−0.0039 (11)	0.0237 (12)	−0.0032 (12)
C26	0.0830 (19)	0.0594 (16)	0.0586 (16)	−0.0087 (14)	0.0230 (14)	0.0026 (13)
C27	0.086 (2)	0.0544 (16)	0.076 (2)	−0.0041 (14)	0.0218 (15)	0.0146 (14)
C28	0.092 (2)	0.0445 (14)	0.080 (2)	−0.0022 (14)	0.0282 (16)	0.0051 (14)
C29	0.0735 (17)	0.0467 (14)	0.0677 (17)	−0.0039 (12)	0.0247 (13)	−0.0053 (13)
C30	0.0488 (13)	0.0450 (13)	0.0584 (15)	−0.0028 (10)	0.0215 (11)	−0.0050 (11)
C11	0.0460 (13)	0.0378 (12)	0.0584 (15)	−0.0020 (10)	0.0204 (11)	0.0011 (10)
C12	0.0445 (13)	0.0455 (13)	0.0551 (14)	−0.0038 (10)	0.0174 (10)	−0.0044 (11)
C7	0.0449 (13)	0.0458 (13)	0.0554 (14)	−0.0010 (10)	0.0155 (11)	0.0037 (11)
C6	0.0494 (14)	0.0566 (15)	0.0575 (16)	−0.0049 (12)	0.0118 (11)	0.0036 (12)
C5	0.091 (2)	0.0721 (19)	0.0633 (19)	−0.0111 (16)	0.0071 (15)	0.0048 (15)
C4	0.109 (3)	0.102 (3)	0.0600 (19)	−0.015 (2)	0.0007 (17)	0.0138 (18)
C3	0.086 (2)	0.087 (2)	0.088 (2)	0.0008 (18)	0.0086 (18)	0.036 (2)
C2	0.0679 (18)	0.0649 (18)	0.086 (2)	0.0024 (14)	0.0151 (15)	0.0206 (16)
C1	0.0469 (14)	0.0537 (15)	0.0702 (17)	0.0007 (11)	0.0162 (12)	0.0091 (13)
C19	0.0531 (14)	0.0442 (13)	0.0538 (14)	−0.0046 (11)	0.0180 (11)	−0.0009 (11)
C20	0.0643 (17)	0.0712 (18)	0.0826 (19)	−0.0174 (14)	0.0358 (14)	−0.0242 (15)
C21	0.0733 (18)	0.0747 (19)	0.0825 (19)	−0.0272 (15)	0.0332 (15)	−0.0247 (16)
C22	0.0803 (19)	0.0528 (15)	0.0639 (17)	−0.0056 (14)	0.0232 (14)	−0.0174 (13)
C23	0.0744 (19)	0.097 (2)	0.092 (2)	−0.0128 (17)	0.0439 (16)	−0.0337 (18)
C24	0.0674 (17)	0.0768 (18)	0.0816 (19)	−0.0217 (14)	0.0348 (15)	−0.0278 (15)
N1	0.0583 (12)	0.0431 (11)	0.0698 (14)	0.0006 (9)	0.0238 (10)	0.0027 (10)
N2	0.105 (2)	0.0823 (18)	0.0870 (19)	−0.0048 (18)	0.0303 (17)	−0.0308 (15)
O5	0.0860 (12)	0.0478 (10)	0.0554 (11)	−0.0062 (8)	0.0271 (9)	−0.0050 (8)
O3	0.132 (2)	0.172 (3)	0.187 (3)	0.000 (2)	0.068 (2)	−0.111 (2)
O4	0.142 (2)	0.0987 (18)	0.1132 (19)	−0.0397 (17)	0.0411 (16)	−0.0519 (15)
S1	0.0718 (16)	0.0251 (13)	0.0596 (17)	0.0034 (10)	0.0278 (11)	0.0003 (10)
O1	0.090 (5)	0.024 (2)	0.106 (4)	0.013 (3)	0.022 (3)	0.011 (2)
O2	0.106 (4)	0.075 (5)	0.049 (3)	−0.002 (3)	0.043 (3)	−0.018 (2)
C13	0.056 (3)	0.041 (3)	0.045 (4)	−0.015 (2)	0.016 (3)	0.000 (3)
C14	0.060 (3)	0.053 (3)	0.043 (3)	−0.007 (2)	0.011 (2)	−0.001 (2)
C15	0.059 (4)	0.086 (4)	0.072 (4)	−0.001 (3)	0.011 (3)	0.011 (3)
C16	0.057 (3)	0.117 (6)	0.089 (4)	−0.014 (3)	0.031 (3)	0.017 (4)
C17	0.077 (4)	0.125 (6)	0.077 (4)	−0.003 (4)	0.038 (3)	0.032 (4)
C18	0.073 (4)	0.087 (4)	0.065 (4)	−0.005 (3)	0.022 (3)	0.029 (3)
S1'	0.083 (4)	0.076 (5)	0.160 (7)	−0.015 (3)	0.058 (4)	−0.027 (4)
O1'	0.098 (9)	0.053 (7)	0.222 (13)	−0.004 (6)	0.014 (7)	−0.009 (6)
O2'	0.142 (10)	0.024 (4)	0.164 (10)	−0.026 (5)	0.110 (8)	−0.031 (5)
C13'	0.084 (8)	0.132 (11)	0.067 (10)	−0.020 (7)	0.027 (7)	0.000 (8)
C14'	0.073 (7)	0.139 (10)	0.075 (8)	−0.020 (6)	0.032 (6)	−0.024 (6)
C15'	0.062 (7)	0.167 (11)	0.110 (9)	−0.015 (7)	0.017 (7)	−0.001 (8)
C16'	0.113 (9)	0.187 (15)	0.153 (11)	−0.019 (9)	0.067 (9)	0.015 (11)
C17'	0.141 (10)	0.178 (14)	0.166 (10)	−0.015 (10)	0.095 (9)	0.024 (11)
C18'	0.119 (8)	0.183 (12)	0.130 (10)	−0.027 (8)	0.061 (8)	0.033 (9)

### (I) 12-(4-Nitrophenyl)-7-phenylsulfonyl-7H-benzofuro[2,3-b]carbazole Geometric parameters (Å, º)

**Table:**

C8—C9	1.381 (3)	C21—H21	0.9300
C8—C7	1.410 (3)	C22—C23	1.368 (4)
C8—N1	1.426 (3)	C22—N2	1.472 (3)
C9—C10	1.363 (3)	C23—C24	1.372 (4)
C9—H9	0.9300	C23—H23	0.9300
C10—O5	1.383 (3)	C24—H24	0.9300
C10—C11	1.390 (3)	N1—S1'	1.645 (6)
C25—O5	1.381 (3)	N1—S1	1.677 (3)
C25—C30	1.381 (3)	N2—O3	1.204 (3)
C25—C26	1.386 (3)	N2—O4	1.209 (3)
C26—C27	1.377 (4)	S1—O1	1.428 (4)
C26—H26	0.9300	S1—O2	1.436 (4)
C27—C28	1.379 (4)	S1—C13	1.740 (3)
C27—H27	0.9300	C13—C14	1.3900
C28—C29	1.374 (3)	C13—C18	1.3900
C28—H28	0.9300	C14—C15	1.3900
C29—C30	1.387 (3)	C14—H14	0.9300
C29—H29	0.9300	C15—C16	1.3900
C30—C11	1.462 (3)	C15—H15	0.9300
C11—C12	1.406 (3)	C16—C17	1.3900
C12—C7	1.403 (3)	C16—H16	0.9300
C12—C19	1.485 (3)	C17—C18	1.3900
C7—C6	1.451 (3)	C17—H17	0.9300
C6—C5	1.390 (4)	C18—H18	0.9300
C6—C1	1.398 (3)	S1'—O2'	1.422 (7)
C5—C4	1.387 (4)	S1'—O1'	1.428 (8)
C5—H5	0.9300	S1'—C13'	1.736 (7)
C4—C3	1.384 (5)	C13'—C14'	1.3900
C4—H4	0.9300	C13'—C18'	1.3900
C3—C2	1.369 (4)	C14'—C15'	1.3900
C3—H3	0.9300	C14'—H14'	0.9300
C2—C1	1.383 (3)	C15'—C16'	1.3900
C2—H2	0.9300	C15'—H15'	0.9300
C1—N1	1.423 (3)	C16'—C17'	1.3900
C19—C24	1.376 (3)	C16'—H16'	0.9300
C19—C20	1.378 (3)	C17'—C18'	1.3900
C20—C21	1.373 (3)	C17'—H17'	0.9300
C20—H20	0.9300	C18'—H18'	0.9300
C21—C22	1.358 (4)		
			
C9—C8—C7	123.5 (2)	C23—C22—N2	119.3 (3)
C9—C8—N1	127.4 (2)	C22—C23—C24	118.6 (2)
C7—C8—N1	109.02 (19)	C22—C23—H23	120.7
C10—C9—C8	114.1 (2)	C24—C23—H23	120.7
C10—C9—H9	123.0	C23—C24—C19	121.2 (2)
C8—C9—H9	123.0	C23—C24—H24	119.4
C9—C10—O5	122.5 (2)	C19—C24—H24	119.4
C9—C10—C11	125.9 (2)	C1—N1—C8	107.16 (18)
O5—C10—C11	111.62 (19)	C1—N1—S1'	122.0 (4)
O5—C25—C30	112.2 (2)	C8—N1—S1'	123.3 (4)
O5—C25—C26	123.2 (2)	C1—N1—S1	120.53 (18)
C30—C25—C26	124.5 (2)	C8—N1—S1	121.89 (19)
C27—C26—C25	115.6 (2)	O3—N2—O4	122.6 (3)
C27—C26—H26	122.2	O3—N2—C22	118.7 (3)
C25—C26—H26	122.2	O4—N2—C22	118.7 (3)
C26—C27—C28	121.1 (3)	C25—O5—C10	105.33 (17)
C26—C27—H27	119.5	O1—S1—O2	120.9 (5)
C28—C27—H27	119.5	O1—S1—N1	109.8 (5)
C29—C28—C27	122.4 (3)	O2—S1—N1	105.2 (4)
C29—C28—H28	118.8	O1—S1—C13	107.3 (5)
C27—C28—H28	118.8	O2—S1—C13	107.5 (5)
C28—C29—C30	118.1 (2)	N1—S1—C13	105.2 (3)
C28—C29—H29	121.0	C14—C13—C18	120.0
C30—C29—H29	121.0	C14—C13—S1	116.5 (4)
C25—C30—C29	118.3 (2)	C18—C13—S1	123.4 (4)
C25—C30—C11	105.33 (19)	C13—C14—C15	120.0
C29—C30—C11	136.3 (2)	C13—C14—H14	120.0
C10—C11—C12	119.5 (2)	C15—C14—H14	120.0
C10—C11—C30	105.46 (19)	C16—C15—C14	120.0
C12—C11—C30	135.0 (2)	C16—C15—H15	120.0
C7—C12—C11	116.4 (2)	C14—C15—H15	120.0
C7—C12—C19	122.4 (2)	C15—C16—C17	120.0
C11—C12—C19	121.1 (2)	C15—C16—H16	120.0
C12—C7—C8	120.5 (2)	C17—C16—H16	120.0
C12—C7—C6	132.6 (2)	C16—C17—C18	120.0
C8—C7—C6	106.8 (2)	C16—C17—H17	120.0
C5—C6—C1	118.6 (2)	C18—C17—H17	120.0
C5—C6—C7	133.2 (2)	C17—C18—C13	120.0
C1—C6—C7	108.0 (2)	C17—C18—H18	120.0
C4—C5—C6	119.2 (3)	C13—C18—H18	120.0
C4—C5—H5	120.4	O2'—S1'—O1'	120.6 (13)
C6—C5—H5	120.4	O2'—S1'—N1	108.6 (7)
C3—C4—C5	120.3 (3)	O1'—S1'—N1	101.5 (11)
C3—C4—H4	119.8	O2'—S1'—C13'	110.4 (12)
C5—C4—H4	119.8	O1'—S1'—C13'	112.2 (12)
C2—C3—C4	121.9 (3)	N1—S1'—C13'	101.3 (6)
C2—C3—H3	119.0	C14'—C13'—C18'	120.0
C4—C3—H3	119.0	C14'—C13'—S1'	125.4 (10)
C3—C2—C1	117.4 (3)	C18'—C13'—S1'	114.5 (10)
C3—C2—H2	121.3	C13'—C14'—C15'	120.0
C1—C2—H2	121.3	C13'—C14'—H14'	120.0
C2—C1—C6	122.6 (3)	C15'—C14'—H14'	120.0
C2—C1—N1	128.4 (2)	C16'—C15'—C14'	120.0
C6—C1—N1	108.9 (2)	C16'—C15'—H15'	120.0
C24—C19—C20	118.2 (2)	C14'—C15'—H15'	120.0
C24—C19—C12	121.1 (2)	C15'—C16'—C17'	120.0
C20—C19—C12	120.7 (2)	C15'—C16'—H16'	120.0
C21—C20—C19	121.5 (2)	C17'—C16'—H16'	120.0
C21—C20—H20	119.2	C18'—C17'—C16'	120.0
C19—C20—H20	119.2	C18'—C17'—H17'	120.0
C22—C21—C20	118.4 (2)	C16'—C17'—H17'	120.0
C22—C21—H21	120.8	C17'—C18'—C13'	120.0
C20—C21—H21	120.8	C17'—C18'—H18'	120.0
C21—C22—C23	122.0 (2)	C13'—C18'—H18'	120.0
C21—C22—N2	118.7 (3)		
			
C7—C8—C9—C10	−0.9 (3)	C6—C1—N1—C8	−0.2 (2)
N1—C8—C9—C10	−177.3 (2)	C2—C1—N1—S1'	32.8 (5)
C8—C9—C10—O5	−178.72 (19)	C6—C1—N1—S1'	−150.8 (4)
C8—C9—C10—C11	0.0 (4)	C2—C1—N1—S1	37.9 (4)
O5—C25—C26—C27	178.4 (2)	C6—C1—N1—S1	−145.7 (2)
C30—C25—C26—C27	−0.9 (4)	C9—C8—N1—C1	175.9 (2)
C25—C26—C27—C28	1.0 (4)	C7—C8—N1—C1	−0.9 (2)
C26—C27—C28—C29	−0.5 (4)	C9—C8—N1—S1'	−33.9 (5)
C27—C28—C29—C30	−0.3 (4)	C7—C8—N1—S1'	149.2 (4)
O5—C25—C30—C29	−179.2 (2)	C9—C8—N1—S1	−39.2 (3)
C26—C25—C30—C29	0.1 (4)	C7—C8—N1—S1	144.0 (2)
O5—C25—C30—C11	−0.4 (3)	C21—C22—N2—O3	172.2 (3)
C26—C25—C30—C11	178.9 (2)	C23—C22—N2—O3	−9.1 (5)
C28—C29—C30—C25	0.5 (4)	C21—C22—N2—O4	−9.4 (4)
C28—C29—C30—C11	−177.8 (2)	C23—C22—N2—O4	169.3 (3)
C9—C10—C11—C12	0.2 (4)	C30—C25—O5—C10	0.9 (2)
O5—C10—C11—C12	178.98 (18)	C26—C25—O5—C10	−178.4 (2)
C9—C10—C11—C30	−177.9 (2)	C9—C10—O5—C25	177.8 (2)
O5—C10—C11—C30	0.9 (2)	C11—C10—O5—C25	−1.1 (2)
C25—C30—C11—C10	−0.3 (2)	C1—N1—S1—O1	−48.8 (5)
C29—C30—C11—C10	178.2 (3)	C8—N1—S1—O1	170.8 (4)
C25—C30—C11—C12	−178.0 (2)	S1'—N1—S1—O1	62 (5)
C29—C30—C11—C12	0.5 (5)	C1—N1—S1—O2	179.7 (5)
C10—C11—C12—C7	0.6 (3)	C8—N1—S1—O2	39.3 (5)
C30—C11—C12—C7	178.0 (2)	S1'—N1—S1—O2	−70 (5)
C10—C11—C12—C19	−177.9 (2)	C1—N1—S1—C13	66.4 (3)
C30—C11—C12—C19	−0.5 (4)	C8—N1—S1—C13	−74.0 (3)
C11—C12—C7—C8	−1.5 (3)	S1'—N1—S1—C13	177 (5)
C19—C12—C7—C8	177.0 (2)	O1—S1—C13—C14	−168.3 (6)
C11—C12—C7—C6	174.7 (2)	O2—S1—C13—C14	−37.0 (5)
C19—C12—C7—C6	−6.8 (4)	N1—S1—C13—C14	74.8 (3)
C9—C8—C7—C12	1.7 (3)	O1—S1—C13—C18	15.2 (7)
N1—C8—C7—C12	178.71 (19)	O2—S1—C13—C18	146.6 (6)
C9—C8—C7—C6	−175.3 (2)	N1—S1—C13—C18	−101.7 (4)
N1—C8—C7—C6	1.7 (2)	C18—C13—C14—C15	0.0
C12—C7—C6—C5	−2.4 (5)	S1—C13—C14—C15	−176.6 (5)
C8—C7—C6—C5	174.2 (3)	C13—C14—C15—C16	0.0
C12—C7—C6—C1	−178.3 (2)	C14—C15—C16—C17	0.0
C8—C7—C6—C1	−1.8 (3)	C15—C16—C17—C18	0.0
C1—C6—C5—C4	−1.2 (4)	C16—C17—C18—C13	0.0
C7—C6—C5—C4	−176.8 (3)	C14—C13—C18—C17	0.0
C6—C5—C4—C3	0.2 (5)	S1—C13—C18—C17	176.3 (5)
C5—C4—C3—C2	0.7 (5)	C1—N1—S1'—O2'	−175.6 (10)
C4—C3—C2—C1	−0.6 (5)	C8—N1—S1'—O2'	38.5 (11)
C3—C2—C1—C6	−0.4 (4)	S1—N1—S1'—O2'	112 (6)
C3—C2—C1—N1	175.6 (2)	C1—N1—S1'—O1'	−47.6 (11)
C5—C6—C1—C2	1.3 (4)	C8—N1—S1'—O1'	166.5 (10)
C7—C6—C1—C2	177.9 (2)	S1—N1—S1'—O1'	−120 (6)
C5—C6—C1—N1	−175.4 (2)	C1—N1—S1'—C13'	68.1 (8)
C7—C6—C1—N1	1.2 (3)	C8—N1—S1'—C13'	−77.8 (8)
C7—C12—C19—C24	−72.6 (3)	S1—N1—S1'—C13'	−4 (5)
C11—C12—C19—C24	105.9 (3)	O2'—S1'—C13'—C14'	−51.1 (13)
C7—C12—C19—C20	109.0 (3)	O1'—S1'—C13'—C14'	171.2 (15)
C11—C12—C19—C20	−72.6 (3)	N1—S1'—C13'—C14'	63.7 (11)
C24—C19—C20—C21	1.0 (4)	O2'—S1'—C13'—C18'	124.6 (12)
C12—C19—C20—C21	179.5 (3)	O1'—S1'—C13'—C18'	−13.0 (17)
C19—C20—C21—C22	−1.5 (4)	N1—S1'—C13'—C18'	−120.5 (9)
C20—C21—C22—C23	0.9 (5)	C18'—C13'—C14'—C15'	0.0
C20—C21—C22—N2	179.5 (3)	S1'—C13'—C14'—C15'	175.5 (12)
C21—C22—C23—C24	0.0 (5)	C13'—C14'—C15'—C16'	0.0
N2—C22—C23—C24	−178.5 (3)	C14'—C15'—C16'—C17'	0.0
C22—C23—C24—C19	−0.5 (5)	C15'—C16'—C17'—C18'	0.0
C20—C19—C24—C23	0.0 (4)	C16'—C17'—C18'—C13'	0.0
C12—C19—C24—C23	−178.5 (3)	C14'—C13'—C18'—C17'	0.0
C2—C1—N1—C8	−176.6 (2)	S1'—C13'—C18'—C17'	−176.0 (11)

### (I) 12-(4-Nitrophenyl)-7-phenylsulfonyl-7H-benzofuro[2,3-b]carbazole Hydrogen-bond geometry (Å, º)

Cg1 is the centroid of the furan ring O5/C10/C11/C25/C30 and Cg3 is the centroid of the benzene ring C1–C6.

**Table:**

*D*—H···*A*	*D*—H	H···*A*	*D*···*A*	*D*—H···*A*
C2—H2···O1	0.93	2.41	3.015 (9)	122
C9—H9···O2	0.93	2.28	2.849 (9)	119
C14—H14···O4^i^	0.93	2.55	3.296 (7)	138
C20—H20···O2^ii^	0.93	2.53	3.454 (11)	172
C16—H16···*Cg*1^iii^	0.93	2.76	3.676 (6)	169
C23—H23···*Cg*3^iv^	0.93	2.99	3.908 (4)	169

Symmetry codes: (i) *x*−1/2, −*y*+5/2, *z*−1/2; (ii) −*x*+1, −*y*+2, −*z*; (iii) −*x*, −*y*+2, −*z*; (iv) −*x*+1/2, *y*+1/2, −*z*+1/2.

### (II) 2-Methyl-4-(4-nitrophenyl)-9-phenylsulfonyl-9H-thieno[2,3-b]carbazole Crystal data

**Table:**

C_27_H_18_N_2_O_4_S_2_	*F*(000) = 1032
*M**_r_* = 498.55	*D*_x_ = 1.413 Mg m^−^^3^
Monoclinic, *P*2_1_/*n*	Mo *K*α radiation, λ = 0.71073 Å
Hall symbol: -P 2yn	Cell parameters from 5100 reflections
*a* = 12.5052 (8) Å	θ = 2.2–27.0°
*b* = 11.2594 (6) Å	µ = 0.27 mm^−^^1^
*c* = 17.0731 (9) Å	*T* = 296 K
β = 102.914 (2)°	Block, colourless
*V* = 2343.1 (2) Å^3^	0.35 × 0.30 × 0.25 mm
*Z* = 4	

### (II) 2-Methyl-4-(4-nitrophenyl)-9-phenylsulfonyl-9H-thieno[2,3-b]carbazole Data collection

**Table:**

Bruker Kappa APEXII CCD diffractometer	5100 independent reflections
Radiation source: fine-focus sealed tube	3714 reflections with *I* > 2σ(*I*)
Graphite monochromator	*R*_int_ = 0.034
ω & φ scans	θ_max_ = 27.0°, θ_min_ = 2.2°
Absorption correction: multi-scan (SADABS; Bruker, 2008)	*h* = −15→15
*T*_min_ = 0.911, *T*_max_ = 0.936	*k* = −14→14
44472 measured reflections	*l* = −21→21

### (II) 2-Methyl-4-(4-nitrophenyl)-9-phenylsulfonyl-9H-thieno[2,3-b]carbazole Refinement

**Table:**

Refinement on *F*^2^	Primary atom site location: structure-invariant direct methods
Least-squares matrix: full	Secondary atom site location: difference Fourier map
*R*[*F*^2^ > 2σ(*F*^2^)] = 0.055	Hydrogen site location: inferred from neighbouring sites
*wR*(*F*^2^) = 0.163	H-atom parameters constrained
*S* = 1.08	*w* = 1/[σ^2^(*F*_o_^2^) + (0.0625*P*)^2^ + 2.4597*P*] where *P* = (*F*_o_^2^ + 2*F*_c_^2^)/3
5100 reflections	(Δ/σ)_max_ = 0.001
317 parameters	Δρ_max_ = 0.44 e Å^−^^3^
0 restraints	Δρ_min_ = −0.36 e Å^−^^3^

### (II) 2-Methyl-4-(4-nitrophenyl)-9-phenylsulfonyl-9H-thieno[2,3-b]carbazole Special details

**Table:**

Geometry. All esds (except the esd in the dihedral angle between two l.s. planes) are estimated using the full covariance matrix. The cell esds are taken into account individually in the estimation of esds in distances, angles and torsion angles; correlations between esds in cell parameters are only used when they are defined by crystal symmetry. An approximate (isotropic) treatment of cell esds is used for estimating esds involving l.s. planes.
Refinement. Refinement of F^2^ against ALL reflections. The weighted R-factor wR and goodness of fit S are based on F^2^, conventional R-factors R are based on F, with F set to zero for negative F^2^. The threshold expression of F^2^ > 2sigma(F^2^) is used only for calculating R-factors(gt) etc. and is not relevant to the choice of reflections for refinement. R-factors based on F^2^ are statistically about twice as large as those based on F, and R- factors based on ALL data will be even larger.

### (II) 2-Methyl-4-(4-nitrophenyl)-9-phenylsulfonyl-9H-thieno[2,3-b]carbazole Fractional atomic coordinates and isotropic or equivalent isotropic displacement parameters (Å^2^)

**Table:**

	*x*	*y*	*z*	*U*_iso_*/*U*_eq_	
C1	0.6060 (2)	0.1940 (2)	0.88312 (17)	0.0422 (6)	
C2	0.5786 (3)	0.2854 (3)	0.8277 (2)	0.0559 (8)	
H2	0.5843	0.3645	0.8438	0.067*	
C3	0.5428 (3)	0.2548 (3)	0.7482 (2)	0.0630 (9)	
H3	0.5242	0.3146	0.7100	0.076*	
C4	0.5337 (3)	0.1379 (3)	0.7237 (2)	0.0631 (9)	
H4	0.5082	0.1203	0.6695	0.076*	
C5	0.5616 (3)	0.0466 (3)	0.77821 (17)	0.0508 (7)	
H5	0.5562	−0.0321	0.7612	0.061*	
C6	0.5983 (2)	0.0746 (2)	0.85962 (16)	0.0390 (6)	
C7	0.6289 (2)	0.0029 (2)	0.93182 (15)	0.0353 (5)	
C8	0.6520 (2)	0.0804 (2)	0.99817 (16)	0.0360 (6)	
C9	0.6768 (2)	0.0413 (2)	1.07625 (16)	0.0393 (6)	
H9	0.6917	0.0937	1.1194	0.047*	
C10	0.6786 (2)	−0.0812 (2)	1.08733 (15)	0.0380 (6)	
C11	0.6568 (2)	−0.1622 (2)	1.02287 (15)	0.0353 (5)	
C12	0.6333 (2)	−0.1197 (2)	0.94342 (15)	0.0350 (5)	
C13	0.8439 (2)	0.2964 (2)	0.98979 (16)	0.0444 (6)	
C14	0.9114 (3)	0.2047 (3)	1.02414 (18)	0.0523 (7)	
H14	0.8886	0.1523	1.0593	0.063*	
C15	1.0124 (3)	0.1910 (4)	1.0062 (2)	0.0681 (9)	
H15	1.0579	0.1287	1.0286	0.082*	
C16	1.0456 (3)	0.2685 (5)	0.9557 (3)	0.0829 (12)	
H16	1.1145	0.2595	0.9443	0.100*	
C17	0.9798 (4)	0.3594 (5)	0.9214 (3)	0.0949 (15)	
H17	1.0039	0.4117	0.8869	0.114*	
C18	0.8765 (3)	0.3742 (4)	0.9378 (2)	0.0735 (11)	
H18	0.8307	0.4354	0.9141	0.088*	
C19	0.6151 (2)	−0.2052 (2)	0.87505 (15)	0.0354 (5)	
C20	0.5183 (2)	−0.2687 (3)	0.85455 (19)	0.0537 (8)	
H20	0.4651	−0.2592	0.8844	0.064*	
C21	0.4998 (3)	−0.3460 (3)	0.7902 (2)	0.0554 (8)	
H21	0.4339	−0.3872	0.7755	0.067*	
C22	0.5802 (2)	−0.3609 (2)	0.74852 (16)	0.0430 (6)	
C23	0.6776 (3)	−0.3019 (3)	0.7681 (2)	0.0584 (8)	
H23	0.7316	−0.3145	0.7393	0.070*	
C24	0.6946 (2)	−0.2227 (3)	0.83158 (19)	0.0531 (8)	
H24	0.7602	−0.1808	0.8452	0.064*	
C25	0.6646 (2)	−0.2867 (2)	1.05076 (15)	0.0384 (6)	
H25	0.6526	−0.3528	1.0173	0.046*	
C26	0.6920 (3)	−0.2909 (2)	1.13271 (18)	0.0481 (7)	
C27	0.7108 (4)	−0.4006 (3)	1.1839 (2)	0.0753 (11)	
H27A	0.6928	−0.4695	1.1504	0.113*	
H27B	0.6651	−0.3981	1.2222	0.113*	
H27C	0.7864	−0.4044	1.2118	0.113*	
N1	0.63886 (18)	0.19995 (19)	0.96867 (14)	0.0411 (5)	
N2	0.5598 (3)	−0.4437 (2)	0.68029 (16)	0.0575 (7)	
O1	0.6654 (2)	0.41840 (18)	0.97873 (17)	0.0737 (7)	
O2	0.7283 (2)	0.29254 (19)	1.09819 (13)	0.0611 (6)	
O3	0.6358 (2)	−0.4681 (3)	0.64953 (16)	0.0853 (9)	
O4	0.4680 (2)	−0.4842 (2)	0.65745 (15)	0.0765 (8)	
S1	0.71501 (6)	0.31228 (6)	1.01429 (5)	0.0488 (2)	
S2	0.70682 (7)	−0.15330 (7)	1.17880 (5)	0.0541 (2)	

### (II) 2-Methyl-4-(4-nitrophenyl)-9-phenylsulfonyl-9H-thieno[2,3-b]carbazole Atomic displacement parameters (Å^2^)

**Table:**

	*U*^11^	*U*^22^	*U*^33^	*U*^12^	*U*^13^	*U*^23^
C1	0.0382 (14)	0.0378 (14)	0.0513 (16)	0.0023 (11)	0.0117 (12)	0.0048 (12)
C2	0.0580 (19)	0.0376 (15)	0.070 (2)	0.0046 (14)	0.0099 (16)	0.0134 (14)
C3	0.068 (2)	0.056 (2)	0.062 (2)	0.0058 (16)	0.0080 (17)	0.0246 (16)
C4	0.076 (2)	0.064 (2)	0.0454 (17)	0.0001 (18)	0.0045 (16)	0.0116 (15)
C5	0.0608 (19)	0.0445 (16)	0.0440 (16)	−0.0026 (14)	0.0049 (14)	0.0012 (13)
C6	0.0371 (14)	0.0362 (13)	0.0443 (15)	−0.0005 (11)	0.0102 (11)	0.0033 (11)
C7	0.0329 (13)	0.0343 (13)	0.0390 (13)	−0.0026 (10)	0.0084 (10)	−0.0021 (10)
C8	0.0336 (13)	0.0288 (12)	0.0467 (15)	−0.0012 (10)	0.0113 (11)	−0.0034 (10)
C9	0.0426 (14)	0.0371 (14)	0.0381 (14)	−0.0032 (11)	0.0088 (11)	−0.0078 (11)
C10	0.0356 (13)	0.0427 (14)	0.0361 (14)	−0.0020 (11)	0.0085 (11)	−0.0002 (11)
C11	0.0311 (13)	0.0346 (13)	0.0407 (14)	−0.0019 (10)	0.0090 (10)	−0.0003 (10)
C12	0.0323 (13)	0.0332 (13)	0.0397 (13)	−0.0031 (10)	0.0087 (10)	−0.0028 (10)
C13	0.0502 (16)	0.0416 (15)	0.0404 (14)	−0.0121 (12)	0.0083 (12)	−0.0014 (12)
C14	0.0515 (18)	0.0549 (18)	0.0494 (17)	−0.0044 (14)	0.0086 (14)	0.0041 (14)
C15	0.0486 (19)	0.083 (3)	0.069 (2)	0.0032 (18)	0.0053 (16)	0.004 (2)
C16	0.053 (2)	0.120 (4)	0.078 (3)	−0.009 (2)	0.0188 (19)	0.008 (3)
C17	0.081 (3)	0.127 (4)	0.083 (3)	−0.025 (3)	0.033 (2)	0.037 (3)
C18	0.068 (2)	0.072 (2)	0.077 (2)	−0.0113 (19)	0.0093 (19)	0.031 (2)
C19	0.0390 (14)	0.0305 (12)	0.0373 (13)	−0.0020 (10)	0.0095 (11)	−0.0005 (10)
C20	0.0455 (16)	0.0565 (18)	0.0640 (19)	−0.0130 (14)	0.0229 (14)	−0.0197 (15)
C21	0.0454 (17)	0.0556 (18)	0.065 (2)	−0.0183 (14)	0.0112 (14)	−0.0174 (15)
C22	0.0514 (16)	0.0332 (13)	0.0427 (15)	0.0001 (12)	0.0073 (12)	−0.0086 (11)
C23	0.0526 (18)	0.067 (2)	0.0615 (19)	−0.0067 (15)	0.0251 (15)	−0.0215 (16)
C24	0.0452 (16)	0.0558 (18)	0.0626 (19)	−0.0183 (14)	0.0215 (14)	−0.0202 (15)
C25	0.0373 (14)	0.0386 (14)	0.0404 (14)	0.0032 (11)	0.0111 (11)	0.0083 (11)
C26	0.0526 (17)	0.0402 (15)	0.0526 (17)	0.0009 (13)	0.0141 (14)	0.0086 (13)
C27	0.107 (3)	0.054 (2)	0.067 (2)	0.007 (2)	0.024 (2)	0.0234 (17)
N1	0.0430 (13)	0.0302 (11)	0.0495 (13)	−0.0007 (9)	0.0090 (10)	−0.0027 (9)
N2	0.0718 (19)	0.0405 (14)	0.0556 (16)	0.0054 (13)	0.0043 (14)	−0.0111 (12)
O1	0.0785 (17)	0.0302 (11)	0.110 (2)	0.0068 (11)	0.0148 (14)	−0.0106 (12)
O2	0.0765 (15)	0.0538 (13)	0.0603 (13)	−0.0182 (11)	0.0305 (11)	−0.0233 (11)
O3	0.091 (2)	0.089 (2)	0.0765 (17)	0.0123 (16)	0.0200 (15)	−0.0388 (15)
O4	0.0852 (19)	0.0571 (15)	0.0761 (17)	−0.0124 (13)	−0.0053 (14)	−0.0266 (12)
S1	0.0555 (5)	0.0302 (3)	0.0627 (5)	−0.0035 (3)	0.0172 (4)	−0.0106 (3)
S2	0.0642 (5)	0.0521 (5)	0.0442 (4)	−0.0033 (4)	0.0081 (4)	0.0038 (3)

### (II) 2-Methyl-4-(4-nitrophenyl)-9-phenylsulfonyl-9H-thieno[2,3-b]carbazole Geometric parameters (Å, º)

**Table:**

C1—C2	1.388 (4)	C15—H15	0.9300
C1—C6	1.400 (4)	C16—C17	1.361 (6)
C1—N1	1.428 (4)	C16—H16	0.9300
C2—C3	1.374 (5)	C17—C18	1.391 (6)
C2—H2	0.9300	C17—H17	0.9300
C3—C4	1.379 (5)	C18—H18	0.9300
C3—H3	0.9300	C19—C24	1.381 (4)
C4—C5	1.378 (4)	C19—C20	1.382 (4)
C4—H4	0.9300	C20—C21	1.381 (4)
C5—C6	1.399 (4)	C20—H20	0.9300
C5—H5	0.9300	C21—C22	1.365 (4)
C6—C7	1.452 (4)	C21—H21	0.9300
C7—C12	1.394 (3)	C22—C23	1.363 (4)
C7—C8	1.408 (3)	C22—N2	1.469 (4)
C8—C9	1.372 (4)	C23—C24	1.383 (4)
C8—N1	1.434 (3)	C23—H23	0.9300
C9—C10	1.391 (4)	C24—H24	0.9300
C9—H9	0.9300	C25—C26	1.365 (4)
C10—C11	1.408 (4)	C25—H25	0.9300
C10—S2	1.725 (3)	C26—C27	1.501 (4)
C11—C12	1.406 (4)	C26—S2	1.729 (3)
C11—C25	1.477 (3)	C27—H27A	0.9600
C12—C19	1.491 (3)	C27—H27B	0.9600
C13—C18	1.372 (4)	C27—H27C	0.9600
C13—C14	1.379 (4)	N1—S1	1.668 (2)
C13—S1	1.762 (3)	N2—O4	1.216 (4)
C14—C15	1.374 (5)	N2—O3	1.216 (4)
C14—H14	0.9300	O1—S1	1.419 (2)
C15—C16	1.356 (6)	O2—S1	1.422 (2)
			
C2—C1—C6	121.7 (3)	C16—C17—H17	119.9
C2—C1—N1	129.3 (3)	C18—C17—H17	119.9
C6—C1—N1	108.9 (2)	C13—C18—C17	118.6 (4)
C3—C2—C1	117.7 (3)	C13—C18—H18	120.7
C3—C2—H2	121.2	C17—C18—H18	120.7
C1—C2—H2	121.2	C24—C19—C20	119.0 (2)
C2—C3—C4	121.7 (3)	C24—C19—C12	120.7 (2)
C2—C3—H3	119.2	C20—C19—C12	120.2 (2)
C4—C3—H3	119.2	C21—C20—C19	120.6 (3)
C5—C4—C3	121.1 (3)	C21—C20—H20	119.7
C5—C4—H4	119.5	C19—C20—H20	119.7
C3—C4—H4	119.5	C22—C21—C20	118.7 (3)
C4—C5—C6	118.7 (3)	C22—C21—H21	120.6
C4—C5—H5	120.7	C20—C21—H21	120.6
C6—C5—H5	120.7	C23—C22—C21	122.3 (3)
C5—C6—C1	119.2 (3)	C23—C22—N2	119.4 (3)
C5—C6—C7	133.1 (3)	C21—C22—N2	118.3 (3)
C1—C6—C7	107.6 (2)	C22—C23—C24	118.6 (3)
C12—C7—C8	120.3 (2)	C22—C23—H23	120.7
C12—C7—C6	131.8 (2)	C24—C23—H23	120.7
C8—C7—C6	107.8 (2)	C19—C24—C23	120.7 (3)
C9—C8—C7	123.0 (2)	C19—C24—H24	119.6
C9—C8—N1	128.7 (2)	C23—C24—H24	119.6
C7—C8—N1	108.2 (2)	C26—C25—C11	110.3 (2)
C8—C9—C10	116.3 (2)	C26—C25—H25	124.9
C8—C9—H9	121.8	C11—C25—H25	124.9
C10—C9—H9	121.8	C25—C26—C27	126.6 (3)
C9—C10—C11	122.8 (2)	C25—C26—S2	114.4 (2)
C9—C10—S2	125.7 (2)	C27—C26—S2	119.1 (2)
C11—C10—S2	111.5 (2)	C26—C27—H27A	109.5
C12—C11—C10	119.7 (2)	C26—C27—H27B	109.5
C12—C11—C25	128.2 (2)	H27A—C27—H27B	109.5
C10—C11—C25	112.1 (2)	C26—C27—H27C	109.5
C7—C12—C11	117.8 (2)	H27A—C27—H27C	109.5
C7—C12—C19	122.3 (2)	H27B—C27—H27C	109.5
C11—C12—C19	119.9 (2)	C1—N1—C8	107.4 (2)
C18—C13—C14	120.7 (3)	C1—N1—S1	121.13 (18)
C18—C13—S1	120.6 (3)	C8—N1—S1	122.65 (18)
C14—C13—S1	118.7 (2)	O4—N2—O3	123.3 (3)
C15—C14—C13	119.7 (3)	O4—N2—C22	118.4 (3)
C15—C14—H14	120.2	O3—N2—C22	118.2 (3)
C13—C14—H14	120.2	O1—S1—O2	120.27 (15)
C16—C15—C14	119.8 (4)	O1—S1—N1	106.83 (14)
C16—C15—H15	120.1	O2—S1—N1	106.12 (12)
C14—C15—H15	120.1	O1—S1—C13	108.62 (15)
C15—C16—C17	121.0 (4)	O2—S1—C13	108.44 (14)
C15—C16—H16	119.5	N1—S1—C13	105.62 (12)
C17—C16—H16	119.5	C10—S2—C26	91.74 (14)
C16—C17—C18	120.2 (4)		
			
C6—C1—C2—C3	0.3 (5)	C7—C12—C19—C20	−106.1 (3)
N1—C1—C2—C3	−175.9 (3)	C11—C12—C19—C20	74.5 (3)
C1—C2—C3—C4	0.2 (5)	C24—C19—C20—C21	−1.8 (5)
C2—C3—C4—C5	−0.8 (6)	C12—C19—C20—C21	178.5 (3)
C3—C4—C5—C6	0.9 (5)	C19—C20—C21—C22	1.7 (5)
C4—C5—C6—C1	−0.3 (4)	C20—C21—C22—C23	−0.3 (5)
C4—C5—C6—C7	176.7 (3)	C20—C21—C22—N2	179.8 (3)
C2—C1—C6—C5	−0.3 (4)	C21—C22—C23—C24	−1.0 (5)
N1—C1—C6—C5	176.7 (2)	N2—C22—C23—C24	178.8 (3)
C2—C1—C6—C7	−178.0 (3)	C20—C19—C24—C23	0.4 (5)
N1—C1—C6—C7	−1.0 (3)	C12—C19—C24—C23	−179.9 (3)
C5—C6—C7—C12	1.2 (5)	C22—C23—C24—C19	1.0 (5)
C1—C6—C7—C12	178.4 (3)	C12—C11—C25—C26	178.4 (3)
C5—C6—C7—C8	−175.8 (3)	C10—C11—C25—C26	−0.4 (3)
C1—C6—C7—C8	1.5 (3)	C11—C25—C26—C27	−178.2 (3)
C12—C7—C8—C9	−1.6 (4)	C11—C25—C26—S2	1.0 (3)
C6—C7—C8—C9	175.7 (2)	C2—C1—N1—C8	176.8 (3)
C12—C7—C8—N1	−178.7 (2)	C6—C1—N1—C8	0.2 (3)
C6—C7—C8—N1	−1.4 (3)	C2—C1—N1—S1	−34.9 (4)
C7—C8—C9—C10	0.1 (4)	C6—C1—N1—S1	148.4 (2)
N1—C8—C9—C10	176.6 (2)	C9—C8—N1—C1	−176.1 (3)
C8—C9—C10—C11	0.3 (4)	C7—C8—N1—C1	0.7 (3)
C8—C9—C10—S2	−179.7 (2)	C9—C8—N1—S1	36.2 (4)
C9—C10—C11—C12	0.7 (4)	C7—C8—N1—S1	−146.90 (19)
S2—C10—C11—C12	−179.24 (19)	C23—C22—N2—O4	−170.9 (3)
C9—C10—C11—C25	179.6 (2)	C21—C22—N2—O4	9.0 (4)
S2—C10—C11—C25	−0.3 (3)	C23—C22—N2—O3	9.1 (4)
C8—C7—C12—C11	2.5 (4)	C21—C22—N2—O3	−171.0 (3)
C6—C7—C12—C11	−174.1 (3)	C1—N1—S1—O1	48.2 (2)
C8—C7—C12—C19	−176.8 (2)	C8—N1—S1—O1	−168.5 (2)
C6—C7—C12—C19	6.6 (4)	C1—N1—S1—O2	177.6 (2)
C10—C11—C12—C7	−2.1 (4)	C8—N1—S1—O2	−39.0 (2)
C25—C11—C12—C7	179.2 (2)	C1—N1—S1—C13	−67.3 (2)
C10—C11—C12—C19	177.3 (2)	C8—N1—S1—C13	76.0 (2)
C25—C11—C12—C19	−1.4 (4)	C18—C13—S1—O1	−5.6 (3)
C18—C13—C14—C15	0.0 (5)	C14—C13—S1—O1	174.2 (2)
S1—C13—C14—C15	−179.7 (3)	C18—C13—S1—O2	−137.9 (3)
C13—C14—C15—C16	0.8 (5)	C14—C13—S1—O2	41.9 (3)
C14—C15—C16—C17	−0.8 (7)	C18—C13—S1—N1	108.7 (3)
C15—C16—C17—C18	0.0 (7)	C14—C13—S1—N1	−71.5 (3)
C14—C13—C18—C17	−0.9 (6)	C9—C10—S2—C26	−179.2 (3)
S1—C13—C18—C17	178.9 (3)	C11—C10—S2—C26	0.8 (2)
C16—C17—C18—C13	0.8 (7)	C25—C26—S2—C10	−1.1 (2)
C7—C12—C19—C24	74.2 (4)	C27—C26—S2—C10	178.2 (3)
C11—C12—C19—C24	−105.2 (3)		

### (II) 2-Methyl-4-(4-nitrophenyl)-9-phenylsulfonyl-9H-thieno[2,3-b]carbazole Hydrogen-bond geometry (Å, º)

Cg1 is the centroid of the thiophene ring S2/C10/C11/C25/C26 and Cg3 is centroid of the benzene ring C1–C6.

**Table:**

*D*—H···*A*	*D*—H	H···*A*	*D*···*A*	*D*—H···*A*
C2—H2···O1	0.93	2.38	2.970 (4)	121
C9—H9···O2	0.93	2.33	2.907 (3)	120
C3—H3···O4^i^	0.93	2.48	3.356 (4)	157
C14—H14···O4^ii^	0.93	2.58	3.335 (4)	139
C20—H20···O2^iii^	0.93	2.53	3.368 (5)	150
C16—H16···*Cg*1^iv^	0.93	2.89	3.812 (4)	172
C23—H23···*Cg*3^v^	0.93	2.75	3.646 (4)	163

Symmetry codes: (i) *x*, *y*+1, *z*; (ii) *x*+1/2, −*y*−1/2, *z*+1/2; (iii) −*x*+1, −*y*, −*z*+2; (iv) −*x*+2, −*y*, −*z*+2; (v) −*x*+3/2, *y*−1/2, −*z*+3/2.
